# Late, but Not Early, Night Sleep Loss Compromises Neuroendocrine Appetite Regulation and the Desire for Food

**DOI:** 10.3390/nu15092035

**Published:** 2023-04-23

**Authors:** Svenja Meyhöfer, Rodrigo Chamorro, Manfred Hallschmid, Denisa Spyra, Nelli Klinsmann, Bernd Schultes, Hendrik Lehnert, Sebastian M. Meyhöfer, Britta Wilms

**Affiliations:** 1 Institute for Endocrinology and Diabetes, University of Lübeck, 23562 Lübeck, Germany; 2 German Center for Diabetes Research (DZD), 85764 Neuherberg, Germany; 3 Department of Internal Medicine 1, Endocrinology & Diabetes, University of Lübeck, 23538 Lübeck, Germany; 4 Center of Brain, Behavior & Metabolism, University of Lübeck, 23562 Lübeck, Germany; 5 Department of Nutrition, Faculty of Medicine, University of Chile, Santiago 8380453, Chile; 6 Department of Medical Psychology and Behavioral Neurobiology, University of Tübingen, 72076 Tübingen, Germany; 7 Institute for Diabetes Research and Metabolic Diseases of the Helmholtz Centre Munich, University of Tübingen (IDM), 72076 Tübingen, Germany; 8 University of Salzburg, A-5020 Salzburg, Austria

**Keywords:** sleep loss, sleep timing, ghrelin, leptin, appetite regulation, healthy men

## Abstract

Objective: There is evidence that reduced sleep duration increases hunger, appetite, and food intake, leading to metabolic diseases, such as type 2 diabetes and obesity. However, the impact of sleep timing, irrespective of its duration and on the regulation of hunger and appetite, is less clear. We aimed to evaluate the impact of sleep loss during the late vs. early part of the night on the regulation of hunger, appetite, and desire for food. Methods: Fifteen normal-weight ([mean ± SEM] body-mass index: 23.3 ± 0.4 kg/m^2^) healthy men were studied in a randomized, balanced, crossover design, including two conditions of sleep loss, i.e., 4 h sleep during the first night-half (‘late-night sleep loss’), 4 h sleep during the second night-half (‘early-night sleep loss’), and a control condition with 8h sleep (‘regular sleep’), respectively. Feelings of hunger and appetite were assessed through visual analogue scales, and plasma ghrelin and leptin were measured from blood samples taken before, during, and after night-time sleep. Results: Ghrelin and feelings of hunger and appetite, as well as the desire for food, were increased after ‘late-night sleep loss’, but not ‘early-night sleep loss’, whereas leptin remained unaffected by the timing of sleep loss. Conclusions: Our data indicate that timing of sleep restriction modulates the effects of acute sleep loss on ghrelin and appetite regulation in healthy men. ‘Late-night sleep loss’ might be a risk factor for metabolic diseases, such as obesity and type 2 diabetes. Thereby, our findings highlight the metabolic relevance of chronobiological sleep timing.

## 1. Introduction

Sleep loss has become common in modern societies [1] and has been reported in different populations and age groups during the past decades [2]. In parallel, the prevalence of obesity and metabolic comorbidities, such as type 2 diabetes (T2D), are rising worldwide [3]. A meta-analysis, including more than 600,000 adults and 30,000 children, showed a strong association between reduced sleep duration of less than 5 h/day and obesity [4]. Large epidemiological studies indicate that reduced quantity and decreased sleep quality are associated with adverse metabolic conditions, such as obesity and impaired glucose tolerance, and several meta-analyses confirmed an association between short, but also long, sleep and the prevalence of the metabolic syndrome [5,6]. An important mediator for these metabolic changes seems to be changes in the central nervous control of energy homeostasis, including an increase in appetite, sensitivity to food stimuli, and energy intake. Thus, sleep loss seems to be a relevant risk factor for metabolic diseases, such as obesity and T2D [7]. As sleep is a process regulated by the circadian clock, being related to the suprachiasmatic nucleus of the hypothalamus as the main zeitgeber and also feedback to the clock [8], the consequences of impaired sleep duration and timing can lead to acute, but also chronic, circadian disruption. In fact, the detrimental health effects of mistimed sleep have been shown by adverse health effects of shift work and social jetlag, two widespread conditions in current societies [9,10,11]. A meta-analysis, including 13 studies, revealed that night shift work is associated with a 57% increased risk of the metabolic syndrome. Data further showed a dose–response relationship with years of night shift work, with 77% increased risk of metabolic syndrome in those with longer exposure to shift work than in those with shorter work in rotating shift (12%) [12].

Most experimental studies have shown that acute sleep loss increases ghrelin and decreases leptin concentrations, as relevant orexigenic and anorexigenic hormonal mediators of hunger and appetite, respectively. In line, short sleep was accompanied by increased feelings of hunger and appetite, as well as caloric intake [13,14], which could promote weight gain and obesity in long term. However, there are also data on unchanged hunger, appetite, and food intake after acute sleep loss [15]. This, at first glance, seems to be a contradictory result, but it may be able to be explained—at least in part—by overeating after both short and regular sleep, since subjects had unlimited access to different foods, including tasty and high caloric items in this last study with ingested energy exceeding the calculated 24 h energy requirement by approximately 60% [15].

The mechanisms linking short sleep to acute changes in feelings of hunger and, thus, shift in the energy balance are under investigation. Elevated ghrelin concentrations might mediate increased food intake, and some, but not all, studies report increased ghrelin concentrations after shortened sleep [16,17,18,19]. In addition, dampened leptin concentrations trigger increased food intake after sleep restriction in some studies [20,21]. However, others have reported unchanged or elevated leptin concentrations after varying degrees of shortened sleep [16,22,23]. Different study protocols could partly explain these contradictory results regarding the degree and duration of sleep restriction, access to food (e.g., unlimited vs. served according to subject’s energy requirement), type of foods (e.g., palatable vs. standard), participants’ characteristics, and physical activity level.

Regarding the desire to eat after short sleep, some studies report no changes in perceived pleasantness of food or the desire to eat after one night with reduced sleep of 4 h and after five consecutive days of insufficient sleep, mimicking a work week, respectively [14,24]. In contrast, others have shown increased hunger and prospective food consumption after three nights of sleep restriction with a sleep duration of 3.5 h per night [25]. Since most studies did not synchronize sleeping and waking periods under experimental conditions, it has been speculated [15] that the waking time is a prerequisite for the orexigenic effect of sleep loss [14,26,27].

The impact of sleep timing, irrespective of its duration and on the regulation of hunger and appetite, is less clear. Alterations in the timing of sleep during restricted sleep could result in dissimilar sleep and metabolic impairments, given the notorious influence of sleep on metabolic processes [7]. Early evidence on this issue showed that mistimed sleep (i.e., early night and late-night sleep loss) during restricted sleep led to changes in sleep architecture in young adults, even without differences in the anorectic hormone leptin levels between sleep restriction-timing conditions [28]. Others have reported that changes in sleep stages after sleep restriction (4 h sleep, from 01:00–05:00 h) was related to changes in hunger feelings [29]. McNeil et al. also showed increased appetite before and after breakfast after short sleep with advanced wake time (i.e., 4 h of sleep with advanced wake up time) in young adults [30]. The same group showed that short sleep with delayed wake-up time increased energy intake from carbohydrates [31]. Additionally, short sleep with both delayed bedtime and advanced wake-up time affected physical activity pattern.

We have previously shown that the timing of sleep loss is relevant for glucose homeostasis and physical activity patterns in healthy humans. Sleep loss, with advanced awakening time, led to reduced glucagon and altered cortisol levels [32] and also led to reduced physical activity and intensity the day after [33]. Thus, besides its consequences on sleep and metabolic outcomes, impairment in the control of appetite regulation can be expected after mistimed sleep loss.

The present study investigated the impact of the specific timing of sleep loss compared to regular sleep on appetite regulation and desire for foods. We hypothesized that both short sleep duration per se and the timing of sleep loss, i.e., early night sleep loss vs. late night sleep loss, may modulate the adverse effects of sleep loss on humans’ appetite regulation, feelings of hunger, and desire for food.

## 2. Methods

### 2.1. Participants

Fifteen healthy (mean ± standard error of the mean [SEM]), normal-weight (BMI: 23.3 ± 0.4 kg/m^2^) young (age: 24.6 ± 0.7 years) male participants were included. This study is part of a larger study investigating the effects of acute sleep loss on glucose homeostasis and physical activity (for details, see [32,33]). Exclusion criteria were chronic or acute illness, current medication of any kind, smoking, elevated alcohol (>50 g per day) and caffeine (>300 mg per day) consumption, diabetes in first-degree relatives, shift work, travel across time zones (past four weeks), and habitual short sleep duration (<6 h per day). Further exclusion criteria were abnormal findings on physical examination or routine laboratory testing (complete blood counts, comprehensive metabolic panel, thyroid function, and lipid composition).

All participants had a regular sleep–wake cycle during the last four weeks before the experiments, with a minimum of 7 h sleep per night. The study protocol was approved by the ethics committee on research, involving humans at the University of Luebeck (Approval code 10-109), in accordance with the Declaration of Helsinki. All participants gave written informed consent before participation.

### 2.2. Study Design and Procedure

Participants were tested in a randomized, balanced, crossover design on three conditions spaced at least three and at maximum five weeks apart. Hormonal orexigenic/anorexigenic balance, subjective feelings of hunger and appetite, general desire for food, as well for savory and sweet foods, were assessed after (i) one night with 4 h of sleep during the first half of the night (‘late-night sleep loss’; bedtime: 22:30 h to 03:00 h), (ii) one night with 4 h of sleep during the second half of the night (‘early-night sleep loss’; bedtime: 02:15 h to 06:45 h), and (iii) one night with regular 8h of sleep (‘regular sleep’; bedtime: 22:15 h to 06:45 h).

For each experimental session, participants attended the research unit at 19:15 h and received a standardized light dinner (380 kcal) at 20:15 h. Thereafter, they were allowed to drink only water (max. 250 mL) until the following day. Participants were then prepared for polysomnographic recordings, and electrodes (C3, C4 derivations) were placed above, below, and beside the eyes for horizontal and vertical electrooculogram. For electromyogram, two electrodes were attached to the chin. Polysomnographic recordings were performed using a Nihon Kohden amplifier (EEG 4400 series, Nihon Kohden GmbH, Rosbach Vor Der Höhe, Germany). Data were scored offline according to standard criteria (for details, see [32,33]). After that, participants went to bed with lights turned off at 22:15 h and 22:30 h, respectively, in the ‘regular sleep’ and ‘late-night sleep loss’ condition. In the ‘early-night sleep loss’ condition, they remained awake in a sitting position until 03:00 h before lights were turned off, and participants were allowed to sleep. Participants were allowed to read and watch non-arousing movies during wake time and were continuously monitored by two experimenters. The light intensity in the laboratory during wake time was 300 lux. In the morning, subjects had no breakfast.

### 2.3. Feelings of Hunger and Appetite

During the wake time in both conditions of sleep restriction, subjective feelings of hunger and appetite, as well as feelings of tiredness and stress, were assessed by a semi-quantitative symptom rating scale scored from 0, i.e., not at all, to 10, i.e., extremely, at 60 min intervals. Likewise, the general desire for foods, as well as the desire for hearty and sweet foods, were assessed using a semi-quantitative symptom rating scale [34]. Ratings were performed using the paper and pencil method.

### 2.4. Blood Samples and Assays

Blood samples were taken after arrival (at 20:00 h), after dinner at 21:00 h, and at 22:00 h. Blood samples were also collected during the night every two hours from 24:00 h to 06:00 h. If under sleep conditions, blood samples were collected via long line running from the participants’ arm to the adjacent room, allowing participants’ sleep not to be disturbed. Subsequent blood samples were taken in the morning at 07:00 h and, thereafter, at 09:00 h, 10:00 h, and 11:00 h until the end of the experiment. Leptin and total ghrelin concentrations were measured by radio-immunoassays (RIA Kit, EMD Millipore, St Saint Louis, MO, USA) with the following within and between assay variation: <8.3% and <6.2% for leptin (mean: 4.9 ng/mL); <10.0% and <14.7% for ghrelin (mean: 1000 pg/mL). All values were determined from stored (−80 °C) plasma samples.

### 2.5. Statistical Analyses

IBM SPSS Statistics, Version 22 for macOS, was used for all analyses, and values are expressed as mean ± SEM. Since values were not always normally distributed at each time point, data were log transformed for leptin and ghrelin and hunger/appetite data and analyzed using analysis of variance (ANOVA) for repeated measures, including the factors ‘condition’ (for ‘early-night sleep loss’ vs. ‘late-night sleep loss’ vs. ‘regular sleep’) and ‘time’ (for repeated measurements during the experiment). In order to correct for sphericity, Green House Geisser correction was applied if necessary. For pairwise comparisons of non-normalized values at single time points, non-parametric tests were applied, i.e., the Friedman test, as well as the Wilcoxon test. The area under the curves (AUC), using the trapezoidal method, were calculated to examine all-over leptin and ghrelin secretion (from 7:00 h to 11:00 h). A *p*-value < 0.05 was considered significant. Power analysis with given alpha (0.05), f (0.4), power (0.8), and correlation among repeated measures (0.6) revealed that 14 subjects are required to obtain significances in addressed parameters.

## 3. Results

Data analyses were performed on 15 subjects, and all of them have finished three visits, including completed data sets on the primary study outcome, i.e., glucose homeostasis, as well as parameters on endocrine hunger regulation.

### 3.1. Sleep

Participants slept 426 ± 43 min in the ‘regular sleep’ condition. As intended by protocol, total sleep time was reduced to 249 ± 18 min and 263 ± 10 min in the ‘late-night sleep loss’ and ‘early-night sleep loss’ condition, respectively (*p* = 0.041 for ANOVA main effect ‘condition’). There was no statistical difference in total sleep time between both sleep loss conditions (*p* = 0.771 for pair-wise comparison; for details, see [32]).

### 3.2. Orexigenic/Anorexigenic Balance

Plasma total ghrelin and leptin concentrations were not different between conditions when participants arrived at the research unit (both *p* > 0.090 for Friedman test).

**Figure 1:**
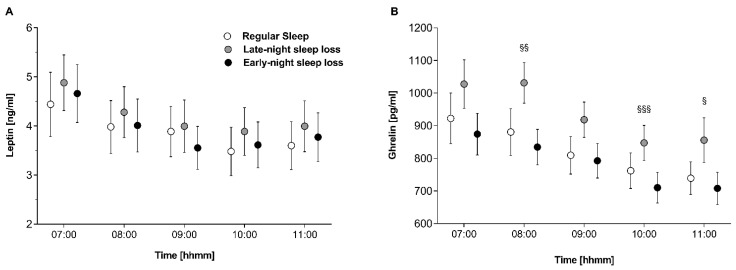
(**A**) Leptin and (**B**) ghrelin concentrations in the morning and during the experimental day after ‘regular sleep’ (open circles), ‘early-night sleep loss’ (black circles), and ‘late-night sleep loss’ (grey circles). ^§^ *p* < 0.05, ^§§^ *p* < 0.01, ^§§§^ *p* < 0.001 for ‘late-night sleep loss’ vs. ’early-night sleep loss’.

Sleep onset (from 22:00 h to 24:00 h for ‘late-night sleep loss’ and ‘regular sleep’; from 02:00 h to 04:00 h for ‘early-night sleep loss’) induced a distinct and rapid increase in leptin concentrations (*p* = 0.031 for ANOVA main effect ‘time’), independent of condition (*p* = 0.812 for ANOVA ‘condition’ × ‘time’ interaction). Analysing of leptin concentrations in the morning at 07:00 h and the end of the experiment (11:00 h) revealed no differences between conditions during the course of leptin concentration measurements (*p* < 0.001 for ANOVA main effect ‘time’; *p* = 0.213 for ANOVA ‘condition’ × ‘time’ interaction; Figure 1A) and the AUC (*p* = 0.32).

Sleep onset was associated with an increase in ghrelin concentrations in the ‘late-night sleep loss’ and ‘regular sleep’ conditions, but not in the ‘early-night sleep loss’ condition (*p* = 0.001 for ANOVA ‘condition’ × ‘time’ interaction). In the morning, at 08:00 h and at the end of the experiment (10:00 h, 11:00 h), ghrelin concentrations were elevated after ‘late-night sleep loss’, as compared to ‘regular sleep’ and ‘early-night sleep loss’ (*p* = 0.004 for ANOVA main effect condition; *p* ≤ 0.008 for pair-wise comparison ‘early night sleep loss’ vs. ‘late-night sleep loss’, Figure 1B). Likewise, the AUCs for ghrelin were different between conditions (*p* = 0.006).

### 3.3. Feelings of Hunger and Appetite

Feelings of hunger and appetite at arrival in the evening were not different between conditions (*p* ≥ 0.557 for Friedman test).

However, feelings of hunger and appetite were markedly increased in the morning after ‘late-night sleep loss’, as compared to ‘regular sleep’ and ‘early-night sleep loss’ (*p* ≤ 0.039 for Friedman test; both *p* ≤ 0.008 for Wilcoxen test ‘late-night sleep loss’ vs. ‘regular sleep’, and ‘late-night sleep loss’ vs. ‘early-night sleep loss’, respectively).

**Figure 2:**
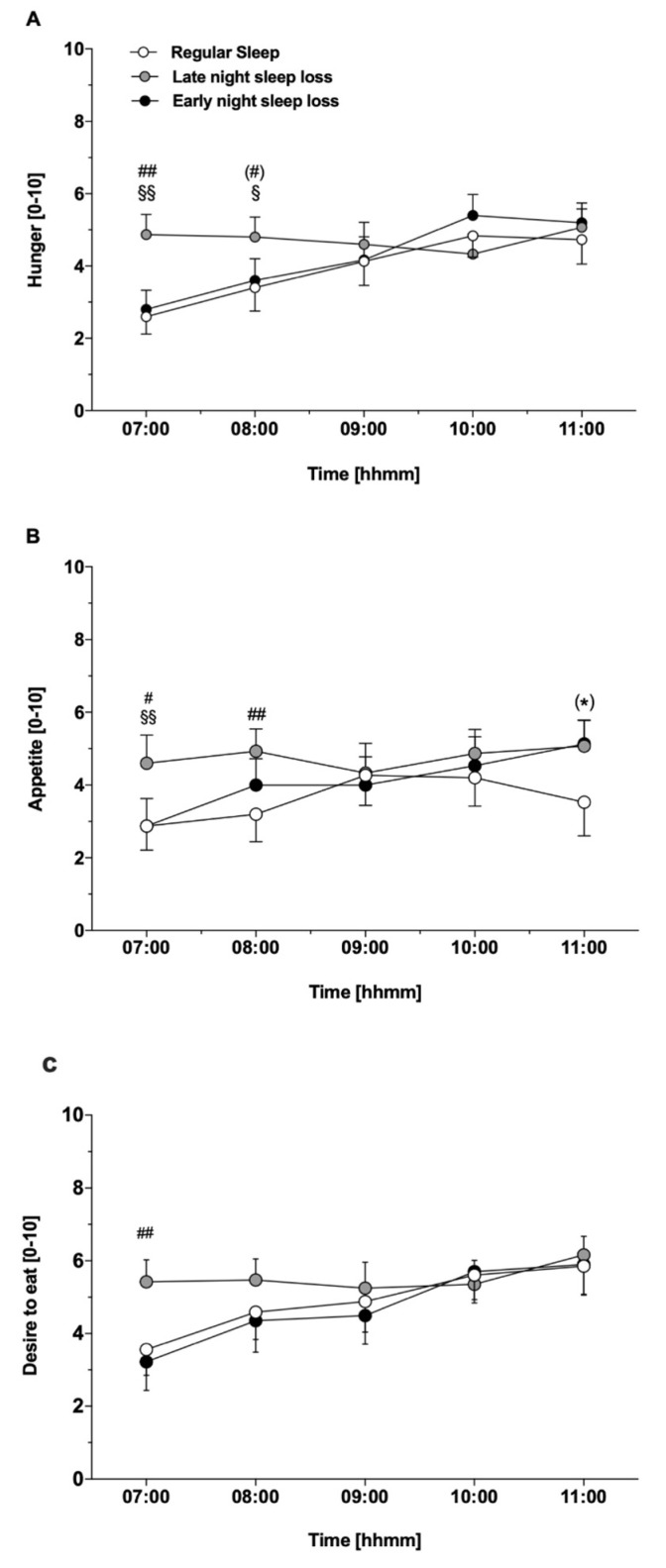
(**A**) Subjective feelings of hunger and (**B**) appetite in the morning and during the experimental day after ‘regular sleep’ (open bars), ‘early night sleep loss’ (black bars), and ‘late night sleep loss’ (grey bars). ^#^ *p* < 0.05, ^##^ *p* < 0.01 for ‘regular sleep’ vs. ‘late night sleep loss’; ^(^*^)^ *p* = 0.05 for ‘regular sleep’ vs. ‘early night sleep loss’; ^§^ *p* < 0.05, ^§§^ *p* < 0.01 for ‘late-night sleep loss’ vs. ‘early-night sleep loss’. (**C**) Desire to eat in the morning and during the experimental day after ‘regular sleep’ (open bars), ‘early night sleep loss’ (black bars), and ‘late night sleep loss’ (grey bars). ^##^ *p* < 0.01 for ‘late-night sleep loss’ vs. ‘regular sleep’ and ‘early-night sleep loss’).

At the end of the experiment (11:00 h), feelings of hunger and appetite converged and were no longer statistically different between conditions (*p* ≥ 0.161 for Friedman test) (Figure 2a,b).

General desire for food at arrival in the evening was comparable between conditions (*p* = 0.936 for Friedman test). In the morning at 07:00 h, there was a significant difference between conditions with highest values in the ‘late-night sleep loss’ as compared to ‘regular sleep’ and ‘early-night sleep loss’ (*p* = 0.001 for ANOVA main effect ‘condition’, Figure 2c). During further course of the experiments, desire to eat converged and failed to be statistically significant (*p* = 0.053 for ANOVA interaction condition × time). This effect was particularly pronounced in the “desire for hearty food” sub-domain (*p* = 0.05 for ANOVA main effect ‘condition’, *p* = 0.038 for pair-wise comparison ‘late-night sleep loss’ vs. ‘early-night sleep loss’). Although there was no general condition effect (*p* = 0.133 for ANOVA main effect ‘condition’), ratings for the sub-domain “desire for sweet food” tended to be higher after ‘late-night sleep loss’ vs. ‘early-night sleep loss’ (*p* = 0.079 for pair-wise comparison). In line with feelings of hunger and appetite, the desire for food was comparable at the end of the experiment (*p* = 0.765 for ANOVA main effect ‘condition’).

## 4. Discussion

In this balanced crossover experiment, we examined the effects of the timing of sleep loss on the regulation of hunger and appetite in humans. ‘Late-night sleep loss’, but not ‘early-night sleep loss’, elevated ghrelin concentrations, as well as feelings of hunger and appetite, and desire for food during the subsequent morning was observed, whereas leptin concentrations were not affected by acute sleep loss per se nor timing of sleep loss. Our data show a more pronounced effect of acute sleep loss on ghrelin, hunger, appetite, and the desire to eat when sleep is restricted in the second half of the night compared to a restriction of sleep during the first half of the night. This underscores the chronobiological role of timed sleep loss in human appetite regulation.

Studies on the effects of sleep restriction (with different timing) on plasma ghrelin and leptin levels have yielded inconsistent results. Our group showed increased ghrelin levels after one night of partial sleep restriction (sleep from 22:30 to 03:30 h) compared to a night with 7 h of sleep [35]. Another study showed that two nights of sleep restriction (sleep from 01:00 to 05:00 h), as compared to two nights with 10 h recovery sleep, resulted in lower leptin and higher ghrelin levels in healthy men, and these changes were associated with increased feelings of hunger and appetite during the subsequent day [20]. Furthermore, four nights of sleep restriction (from 01:00 to 05:30 h) increased ghrelin levels with no changes in leptin, and the increase in ghrelin levels correlated with ingested calories from sweet foods [17]. Overall, most, but not all [18] data, seem to be aligned with our study, which shows elevated ghrelin levels when sleep loss occurs in the late part of the night. With regard to the measurement of ghrelin levels within the different studies, one needs to point out that some studies reported on total ghrelin, whereas others reported on acylated and deacylated ghrelin, separately, which would have been more informative. In some studies, changes in ghrelin were also correlated with increased energy intake [17,20], supporting altered homeostatic control of appetite after sleep loss during the second half of the night. Broussard et al. reported on increased ghrelin levels and caloric intake by more than 300 kcal after four nights of 4.5 h sleep compared to four nights of 8.5 h sleep in lean men. Furthermore, elevated ghrelin levels in the evening were related to higher energy intake, mainly due to carbohydrates (12). Besides ghrelin and leptin, measurements of orexigenic and anorexigenic adipokines, such as Peptide YY (PYY) or Glucagon-like peptide 1 (GLP1), would be relevant to comprehensively assess the effects of timed sleep loss on neuroendocrine appetite regulation.

We showed elevated feelings of hunger and appetite, as well as desire for food, when sleep was restricted, again, in the late part of the night. McNeil et al. evaluated the effects of shortened sleep (~4 h) using an advanced wake time (similar to our ‘late sleep loss’ condition) vs. a delayed bedtime condition (similar to our ‘early sleep loss’ condition) in 18 healthy young men and women [36]. They reported higher fasting appetite and post-breakfast ratings after short sleep with the advanced wake time. Additionally, increased explicit wanting and liking for high-fat relative to low-fat foods after short sleep was observed with advanced wake time vs. the control sleep condition [30]. Another study reported increased energy intake and pre-prandial hunger levels upon one night of 4 h sleep restriction during the second half of the night (from 02:00–06:00 h), as compared to 8 h of regular sleep [14].

Interestingly, a 3.5 h sleep condition using an early night sleep loss protocol (wake until 03:30 h) for three days in healthy men increased hunger and prospective food consumption, as reported by Hibi and co-workers [25]. These results contrast with previous findings from our group, showing that two nights of sleep loss during the first half of the night (wake until 02:45 h) did not affect feelings of hunger and appetite and energy intake in healthy humans [15]. In line with this, our present data also disagree with the study by Hibi et al., as early night sleep loss did not affect ghrelin or hunger feelings compared with late night sleep loss or a control sleep condition. This discrepancy could be related—at least partly—to the different study duration, one and two nights, respectively, in our studies vs. three nights in the study by Hibi and co-workers. It is also worth mentioning that it has been proposed that—in Westernized settings—the assessment of hunger and appetite feelings would reflect the hedonic component, rather than the homeostatic regulation of food intake [36]. Our data showed increased hunger and appetite feelings in the morning (mainly after late night sleep loss) could relate to exacerbated hedonic component of appetite control in the morning after both sleep loss conditions. However, the increased morning ghrelin levels could reflect the adverse effect of a late-night sleep loss for the homeostatic component of energy intake regulation.

Other have also shown increased explicit wanting and liking for high-fat relative to low-fat foods after short sleep with advanced wake time vs. a control sleep condition [30]. Liking and wanting for foods are two separate processes modulating food reward [37]. Whereas liking mirrors the sensory pleasure experience, wanting reflects the motivation related to appetite and, putting forward, the actual desire for food reward. Cerebral structures are differently involved in appetite control, as shown by functional imaging studies. Wanting-related processes are related to ventral pallidum and striatum, whereas liking specific regions are the orbitofrontal cortex, insular cortex, and amygdala, which hints at a neural dissociation of liking and wanting for foods and food-related cues [38]. When addressing the wanting/liking process in the context of sleep restriction, it is relevant to consider that time of day impacts wanting, but not liking, for food. We could show that, both under free living conditions and laboratory settings, liking for energy-dense foods was not different in the morning and evening, whereas wanting was clearly increased in the evening, albeit feelings of hunger and satiety remained unchanged [39]. In our present study, however, liking and wanting were not addressed, which would have allowed for a closer insight into the neuroendocrine appetite regulation, especially in the context of timed sleep loss.

Chronic circadian disruption not on only occurs during insufficient and short sleep, but also during rotating shift work or (social) jetlag and, thus, affects neuroendocrine appetite regulation. Social jetlag is a common form of circadian rhythm disruption and presents in up to 70% of the population [9]. It is defined as the misalignment between individual’s endogenous circadian clock and the actual sleep time [40]. One cross-sectional observational study reported elevated appetite for caloric-dense food in subjects with social jetlag, independent of sleep deprivation compared with subjects with sleep deprivation alone [41]. In addition, ghrelin levels were elevated in those with social jet lag. These data point to a social jetlag-related increase in incentive value of food, together with anticipated pleasure of ingesting these foods. Upon a fMRI study, the same workgroup reported that social jetlag is associated with altered resting-state activity in brain regions related to hedonic feeding, i.e., the reward system. In detail, an increased activity in the putamen, part of the striatum with functions linked to the reward system, was shown in subjects with social jetlag. Importantly, this effect is independent of short sleep duration. Further, subjects with social jetlag reported a higher perceived appetite for high caloric food and had overall detrimental eating habits than those without social jetlag [42]. Thus, data support evidence that subjects with social jetlag are prone to hedonic feeding and, in the long term, to body mass gain.

Under an acute sleep restriction setting, experimental studies report on increased caloric intake due to night-time calories. On the other hand, it has been shown, both in mice and humans, that feeding either at normal eating time while performing a rotating shift work paradigm [43], or having caloric intake matched for the caloric needs while under chronic sleep restriction [44], did not alter circadian rhythms. In the latter study, subjects were exposed to a 32-day inpatient protocol, including 24 cycles of a 20 h forced desynchrony protocol either with or with sleep restriction. There was no impact of short sleep in subjective feelings of hunger that was detectable because hunger rhythm remained stable with a peak in the evening and low levels in the early morning, as already shown by Scheer et al. [45]. Chronic circadian disruption, however, was associated with decreased subjective feelings of hunger, independent of the sleep duration. This study provides deeper insight into the different contributions of chronic circadian disruption per se and short sleep and suggested a less strong impact of short sleep on subjective hunger than reported by some studies [20,35], but not all [15]. Further studies are needed here to evaluate the distinct effects of timing of sleep loss in the context of chronic circadian disruption, since one may speculate that the chronobiological aspects of short sleep differently—at least in part—affects feeling of hunger, as shown in our present study.

We assessed appetite regulation and desire for food after only one night of sleep restriction by four hours compared to control sleep of 8 h. Therefore, we cannot draw any conclusions concerning the effects of longer-lasting sleep restriction or, on the other hand, shifted sleep, as well as social jetlag. Further studies are needed to evaluate the prolonged effects of sleep restriction with altered sleep timing, e.g., using a forced desynchrony protocol [44]. However, the possibility to perform such studies in humans under experimental settings is limited compared to conducting an animal study using a shift work paradigm (e.g.,). As a further limitation, our study population included young, healthy, and normal-weight men. Although we do not expect gender differences, we cannot extrapolate the observed effects on women. In fact, current evidence regarding the metabolic consequences of sleep restriction has been provided from studies conducted mostly in men. However, studies comprising young healthy adults, both men and women, have reported consistent increases in hunger, appetite, energy intake, and susceptibility to food stimuli [46], together with reduced insulin sensitivity, in young adults [19]. Importantly, those studies have used different duration of sleep loss intervention. In females, increased morning leptin levels [22,47], but no changes in hunger scores, have been shown after an acute partial sleep restriction (3 h of sleep) [22]. With longer duration of intervention (four nights), increased energy intake and weight gain have been reported after partial sleep restriction in healthy women [13]. Others, comparing the effects of sleep loss in men and women, have shown increased ghrelin levels after four days of sleep restricted to 4-h only in men [48]. Additionally, after five days of 4 h of sleep (sleep from 04:00 to 08:00 h), men, but not women, showed exacerbated overall and late-night hour energy intake [49]. These last studies, however, are not comparable to our current study, as we assessed a very acute (one night) sleep loss intervention. It is further important to point out that, despite the small number of participants, a parametric test procedure was used. To address sphericity, we used the Green House Geisser approach. Moreover, our results should be confirmed in subjects prone to metabolic diseases, such patients as with obesity and T2D. As discussed above, using more comprehensive methods to assess the regulation of food intake, such as the wanting/liking task, or fMRI, would provide deeper insight into the impact of short sleep and its timing

## 5. Conclusions

Altogether, our data clearly show that chronobiological aspects modulate the detrimental effects of sleep loss on the regulation of food intake. Therefore, our results could be of clinical interest regarding sleep hygiene and appropriate sleep recommendations in our 24 h society with commonly curtailed sleep duration. However, one should always consider the small sample size. In conclusion, concerning the background of the well-known association between shortened sleep duration and increased risk for developing obesity and T2D, furthers studies are required to figure out the impact of timing of short sleep in wider groups of participants and under non-laboratory settings.

## Data Availability

The data presented in this study are available on request from the corresponding author. The data are not publicly available due to ethical restriction.
